# Nature, Calcigender, Nurture: Sex-dependent differential Ca^2+^ homeostasis as the undervalued third pillar

**DOI:** 10.1080/19420889.2019.1592419

**Published:** 2019-04-02

**Authors:** Arnold De Loof

**Affiliations:** Functional Genomics and Proteomics Group, Department of Biology, KU Leuven-University of Leuven, Leuven, Belgium

**Keywords:** Sex-steroids, cultural evolution, social psychology, nature versus Nurture, gender, gender neutrality, masculinism, feminism, transgender

## Abstract

After many years of sometimes heated discussions, the problem regarding the relative importance of two classical dogmas of the Nature (genes and sex-steroid hormones) versus Nurture (education, teaching-learning etc.) debate, is still awaiting a conclusive solution. Males and females differ in only a few (primordial) genes as is well documented by genomic analyses. However, their sex- and gender-specific behavior and physiology is nevertheless profoundly different, even if they grew up in a similar (educational) environment. By extending the “Calcigender-concept”, originally formulated in 2015, to the simplistic binary Nature versus Nurture concept, a novel framework showing that the sex-steroid hormone-dependent intracellular Calcium concentration is an important third factor may emerge. Although the principles of animal physiology and evolution strongly stress the fact that Nature is always dominant, Nurture can, to a limited extent, play a mitigating role.

## Introduction

The long-running “Nature versus Nurture” debate is about whether human behavior is mainly determined by the person’s genes (DNA) or by his/her social/educational environment, either prenatal or/and during the person’s life. To date, the not yet generally accepted consensus is that it is not “or” but that both play a role. The key issue is the relative importance of Nature and Nurture: more or less equal or a dominance of one of them, in particular of “Nature”? Despite all progress in the genetics of sex determination, in (neuro)physiology and in sociological gender studies, some questions remain unanswered. Why is male-female physiology and even more their behavior so different while males and females only differ in relatively few genes? The explanation is that not so much different sex-specific genes, but rather differential gene expressions may cause the differences. A second problem is why there are only two genetic sexes, but several gender forms.[1] Another problem is our rudimentary knowledge about the functioning of our cognitive memory system at the molecular level, particularly about the role of self-generated electricity, electrical signaling, and the plasma-membrane- cytoskeletal complex in this process[2]. It is well documented that sex-steroids play a major role in reproduction-related behavior. Yet, it is difficult to explain why there are only two types of gonads, two families of sex-steroids that are mainly secreted by the gonads, namely androgens and estrogens, that structurally these steroids are not drastically different, but that nevertheless there are more than two gender forms The mainly social implications of the cited questions come together in the questions: *To what extent is* Homo developed *through Nurture? If it is feasible at all, is such development desirable, and to what extent?*

The intrinsic problem here is that the mode of action of sex-steroids is well documented at the level of action via nuclear receptors-transcription factors (= genomic effects), but much less at the level of the functional role of the cell membranes. These membranes harbor various enzymes that are involved in generating non-genomic effects, e.g. in lipid and steroid biosynthesis, osmoregulation, and even more important in Ca^2+^-homeostasis. It is possible that in the past too much emphasis may have been given to the intranuclear/genetic mode of action of sex-steroids, neglecting nongenomic effects. There are major differences in Ca^2+^-metabolism and homeostasis between males and females. Furthermore, Ca^2+^ plays a key role in controlling muscle contraction as instrumental to behavior and gamete production. Why was the role of differential Ca^2+^-homeostasis on gender-linked behavior not considered?[1]

## To date’s sexual reproductive physiology of animals has ancient roots in evolutionary history

Researchers in the biomedical sciences versus in the humanities often have a different approach towards the Nature-Nurture debate. In particular, the retrograde timescale differs. In the humanities, gender equality and the means to realize it ever better are a recent key issue. Sex-related genetic differences and differential Ca^2+^-homeostasis are not manipulable, are thus not at the center of their research interest. On the other hand, animal physiologists have a much longer retrograde perspective, but their approach little touches human sociology. They ask questions about how sexual reproduction started at a time in which reproduction was asexual, thus without egg- and sperm cells (gametes), using the principles and mechanisms of regeneration. How the mode of reproduction changed from asexual towards sexual in placental mammals is a fascinating detective story with unexpected plot turns[3]. Some key biochemical signaling pathways have been preserved for many millions of years in all animal species. Others came into being more recently. In the context of signaling, the importance of homeostasis during evolution is a central issue[4].

Whether males and females can or/and should be forced to become more gender-alike in a heterosexual relation mode is only an issue in the species *Homo sapiens*. No other animal species is known to encourage such convergence. In all non-human animal species, the rule is: A male is a male, and a female is a female: the genetics of sex dominate. Apparently, in the animal Kingdom as a whole fitness of the species seems to be better served by the differences in gender than by the similarities. But *Homo sapiens* has a superior cognitive memory system that enabled him/her to realize technical improvements in living conditions and in fitness so that not every member of the group/population had to be engaged in food acquisition, care, and protection. New jobs came into being, some of which could, in theory, be done by both males and females, irrespective of their value for reproductive fitness of the population. Gender-competition came into being, in particular for jobs in which muscular strength matters less than cognitive capabilities. This triggered discussions about the relative importance of the genetic memory system (DNA → RNA → Proteins) versus the cognitive memory system. Herein self-generated electrical pulses carried by inorganic ions play a crucial role [2,5], but despite all progress, this memory type continues to be a largely black box. A challenging question is whether both memory systems act independently of each other, or whether they can influence and even change each other, so that the final outcome of this mutual influence is that gender-inequality (in humans) can be manipulated into (more) gender-neutrality. More precisely, for biologists the question is: During hundreds of million years, very well-conserved signaling pathways causal to sexual reproduction, did not show a drive towards realizing male-female behavioral equality, on the contrary. Is it then realistic to think that this classical male-female binary system can be remodeled in only a few human generations, without interfering in the biochemical signaling pathways, thus only by changes in Nurture?

## Do gender and sexual reproduction have an (evolutionary) goal?

The answer by many people to this question is: Of course, because the ultimate goal is to produce a progeny. Yet, this at first sight self-evident and logical reply is in conflict with a basic rule in evolutionary theory that says that there is no goal whatsoever in evolution, although some recent experimental data suggest that in some circumstances, it may be possible[6]. Long ago, the formation of egg- and sperm cells did not result from planning, but from unplanned mutations. Rather, it was the accidental result of the coming into existence of “aberrant stem cells of the germ cell line” against which the somatic cells of the body developed an (immunological) rejection strategy[3]. Because it failed to kill the growing cells of the germ cell line early in their development, they kept growing (the oocytes in particular) or/and multiplying (in particular the sperm cells). At the end ejection of the gametes from their production sites (ovary and testis) and even of a baby as in humans and other placental mammals, was the only option left for the producing individuals to survive. This contrasts with our belief that producing gametes and a progeny is very good because it increases fitness and assures the continuation of the population. However, from the physiological point of view, being a male or a female indicates a (disease) state controlled by toxic Ca^2+^-levels. [1,3,7]

One should also keep in mind that probably both females and males of most animal species do not know that having heterosexual sex is causal to the production of a progeny. For them, a progeny is an unexpected free bonus when having engaged in hormone-driven copulation behavior. Having sex is a stronger drive than producing a progeny. This is an important issue in the discussion about “Sex versus Gender”. [1]

## Reminder of a few well-established key genetic and physiological principles

### Genetics of sex determination. the human Y chromosome

**Figure 1.**
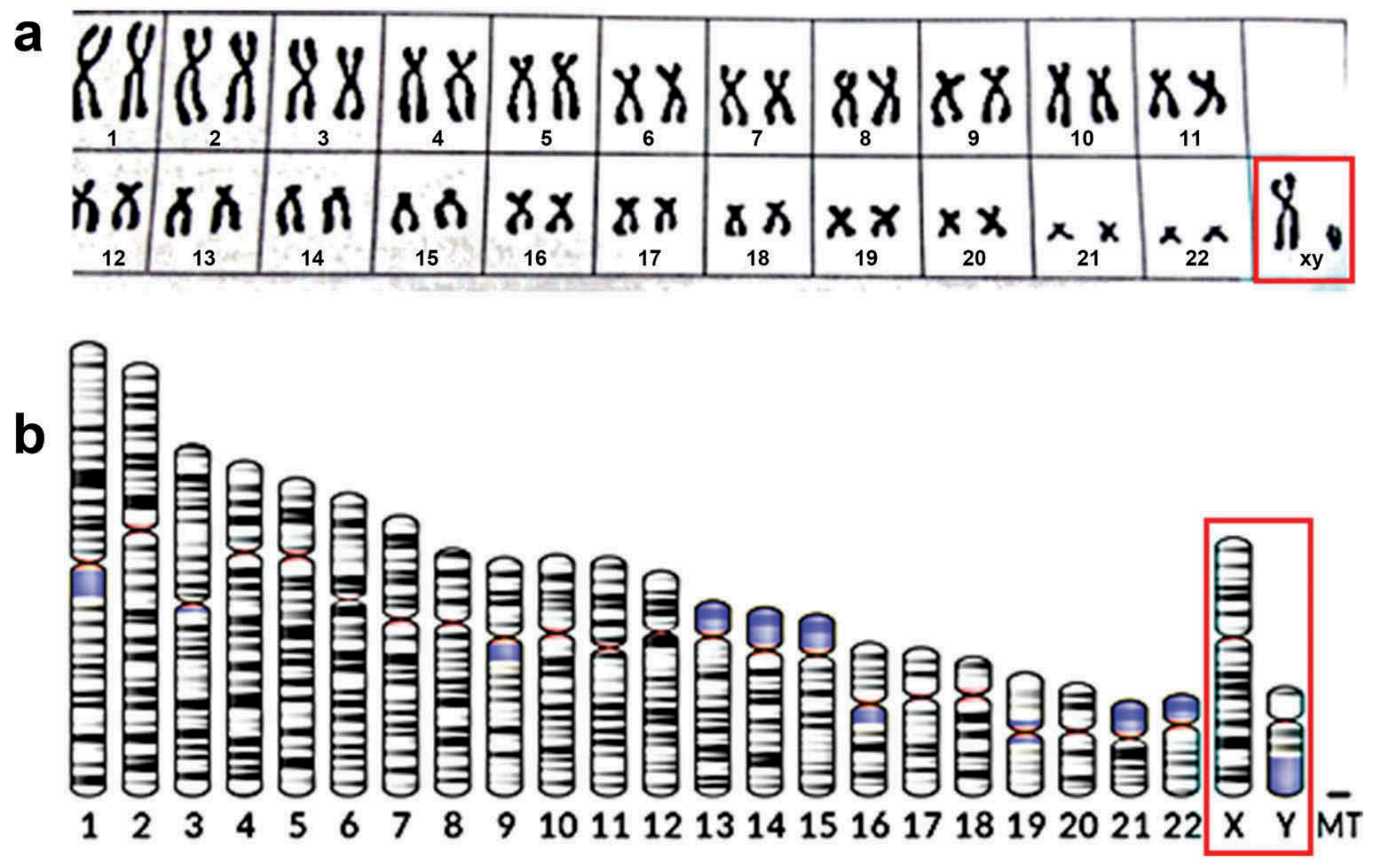
a. General human karyotype. Author: Raj.paljun13 Own work (Created 11/03/2016). Background color has been made less dark than in original. Copyright permission: freely accessible under creative Commons (CC BY-SA 4.0). With thanks to the author. From Wikipedia: Karyotype (human male). [9] b. Ideogram of human chromosome. Chromosome Y highlighted. G-band, 850 bphs (bands per haploid set). Black and gray: Giemsa positive. Red: Centromere. Light blue: Variable region. Dark blue: Stalk National Center for Biotechnology Information, U.S. National Library of Medicine – File created 29 July 2017 NCBI’s Genome Decoration Page. From Wikipedia: Testis Determining Factor. [10] Copyright: Public domain, with thanks. X: X chromosome; Y: Y chromosome; MT: mitochondrial.

Diploid cells of the species *Homo sapiens* have 46 chromosomes, of which 44 are autosomes that occur in both males and females, and two are sex chromosomes (XX in females and XY in males) (Figure 1). For the figure of human male karyogram see Wikipedia: Y chromosome. [8] The form of the sex chromosomes by themselves is not important. In birds e.g., the configuration is ZZ (males) and ZW (females). In the fruit fly *Drosophila melanogaster*, males have one chromosome less than females, and this is indicated as XO for males and XX for females. For more details and for mechanisms of sex-determination in other species, in particular, non-mammalian species, see textbooks of Developmental Biology. Their variability is high.

Yet, the reproductive physiology of non-mammalian species is in many aspects similar to that of placental mammals. As will be outlined later, despite some differences in mechanisms, the common physiological outcome is “differential sex-dependent Ca^2+^ homeostasis” which enables females to secrete more Ca^2+^ than males, e.g. in yolk-rich eggs and in milk of mammals. Such ability implies that the cells involved in the secretion of high amounts of Ca^2+^ through the production and secretion of Ca^2+^-transporting proteins use such mechanism to cope with the problem of the toxicity of high intracellular Ca^2+^ concentrations[7].

In most mammals, the Y chromosome is very important in sex determination. Its presence is dominant over that of the X chromosome. The human X chromosome spans approximately 58 million base pairs which corresponds to about 1% of the total DNA in a male cell (Wikipedia: Y chromosome). It carries about 900–1600 genes. The much smaller human Y chromosome carries over 200 genes, at least 72 of which code for proteins. The question is: Out of these 200+ genes which primordial inducer(s) trigger(s) the sex-determining cascade? Here only the system in the species *Homo sapiens* is discussed in which the well-documented Testis-Determining Factor (TDF), also known are SRY gene/protein, is of utmost importance (see later).

### *Male humans and most but not all mammals have a male-determining gene*, SRY, *located on the Y chromosome*

**Figure 2.**
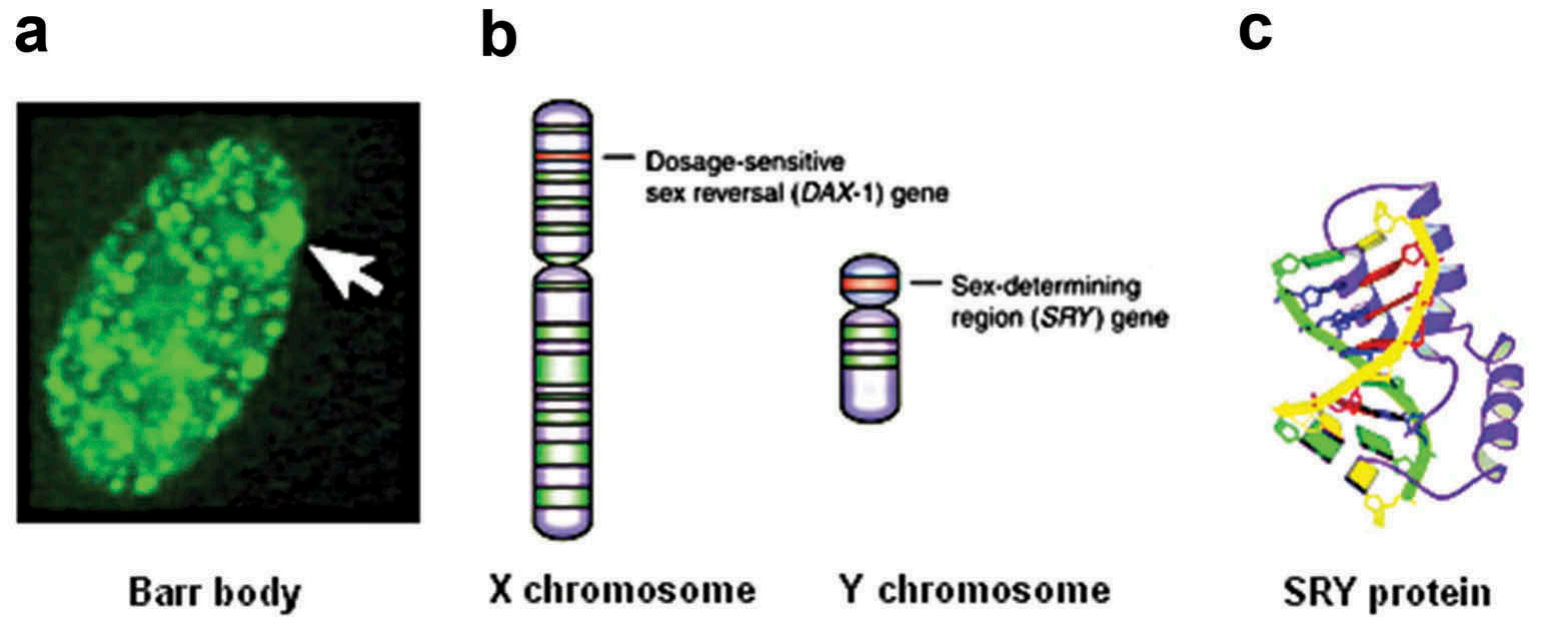
a. Barr body, the condensed second X chromosome[17]. Photomicrograph example of normal fibroblast that was FITC-labeled using antisera to histone macroH2A1. Arrow points to sex chromatin site in the FITC-labelled photo. This file, originally published by Gartler et al. [12,13], is licensed under the Creative Commons Attribution 2.0 Generic license. With thanks. b. Location of genes involved in gonadal sex differentiation. The sex-determining region of the Y (*SRY*) gene codes for the production of the SRY protein, which causes testis differentiation. Absence of this gene in an individual lacking the Y chromosome results in the formation of ovaries. The *DAX-1* gene on the X chromosome suppresses *SRY* gene expression in a rather complex way. Authors: Jones and Lopez 17 with thanks. Copyright permission obtained from Elsevier. c. Image of the SRY protein (in violet) partially inserted in between two DNA strands (in green and yellow). From Wikipedia: Testis-determining factor.10 Copyright permission details: File:PBB Protein SRY image.jpg, Uploaded: 3 January 2010: stated to be public domain from www.pdb.org. With thanks to the non-disclosed author.

There are so many differences in the morphology, physiology, and behavior of male and female animals in general that one is tempted to think that males and females must differ in many genes. If only mammals are considered, this idea is wrong. In the majority of mammals, namely placental mammals, and the marsupials, the difference-making situation is that, compared to females, males have one extra gene, named *SRY* (Wikipedia: Testis determining factor)[10]. This intronless gene is located on the short arm of Y (Figure 2a) (details in: Ensemble, May 2017)[11]. It codes for the SRY protein (Figure 2b) which is also named Testis Determining Factor (TDF)[10]. In addition, many other genes which are present in both sexes, are differentially expressed during development, in particular under the influence of sex-steroid hormones. The most “primitive” group of mammals, the Monotremes which lay eggs like reptiles and birds do, but which produce milk and suckle their young like the other mammals, have no TDF. It is thought that after the split between the Monotremes and the therians (= marsupials and placental mammals,) the SRY gene may have arisen from a gene duplication of the X chromosome-bound gene *SOX3*, a member of the Sox family. If the *SRY* gene is active, the fertilized egg (zygote) will develop into a male. At first sight, the presence of an extra gene (*SRY*) in most male mammals, may seem to be a good argument for stating that males are genetically superior compared to females. But females have 2 X chromosomes while males have only one X, thus in this aspect females are genetically superior. However, when one takes into account that in females one of the two X chromosomes gets inactivated into a Barr body (see later) early in development, the assumed female superiority vanishes. What really matters is the physiological outcome of the genetic basis of sex determination. Here, the fact that females live longer than males in most species leads to the conclusion that in some aspects females are physiologically superior compared to males.

### Mode of action of the testis-determining factor

The SRY/TDF protein acts as the primordial inducer that triggers many other genes into a male-generating activity. [14–16] It is active in the nucleus as a transcription factor. To be functional in sex determination, the SRY/TDF protein needs complexation with other transcription factors, in particular SOX9. The resulting protein complex activates still another factor. This initiates a succession of developing structures such as the primary sex cords, the seminiferous tubules, and part of the undifferentiated gonad, turning it into a testis. Further inductions result in the formation of the Leydig cells, which will start secreting testosterone. The Sertoli cells will produce anti-Müllerian hormone. The combination of these hormones inhibits the female anatomical structural growth in males. It also promotes male dominant development. [10]

### XX chromosomal configuration in women. the barr body

As already mentioned in brief before, a diploid chromosome configuration is generally assumed to be better than a haploid one in the event one of the homologous genes does not work properly. Hence, if there are no restrictions, the mammalian female XX configuration could be considered as genetically superior. However, this assumption fails to take into account the peculiar and exceptional mechanism of random inactivation of one of the X chromosomes in all somatic cells, a process that takes place early in development. The inactivated X chromosome remains (temporarily) active for 10–15%, which corresponds to about the number of active genes present on the Y chromosome. Later, the inactive X chromosome remains microscopically visible as a “Barr body” [17] which is attached to the inner side of the nuclear envelope. A Barr body (named after its discoverer Murray Barr) is not only found exclusively in *Homo sapiens*. It is also visible in the nucleus of those species in which sex is determined by the presence of the Y or the W chromosome rather than by a diploid X chromosome. The process of inactivation of one of the X chromosomes is known as “Lyonization”. [18]

Thus, functionally, females are for most of their life haploid for their sex chromosomes. If one takes this situation into account, males who have also one X but in addition a Y chromosome, have the genetically superior sex form. This does not a priori result in a “better physiological condition” in males. It is well documented and known in many animal species that females live longer than males.

In summary: males of most mammalian species have a testis-determining factor that is absent in females. Although females are diploid for the X chromosome (XX), they only have one X that is active because the second X is randomly inactivated in all somatic cells of the body. Such females are in fact a mixture of two genetically different individuals (nearly identical twins). None of these genetic differences is under the control of Nurture.

## Sex-steroid hormones

### *Testosterone as an* anabolic *steroid and estrogens-estradiol as* lipogenic *steroids*

**Figure 3.**
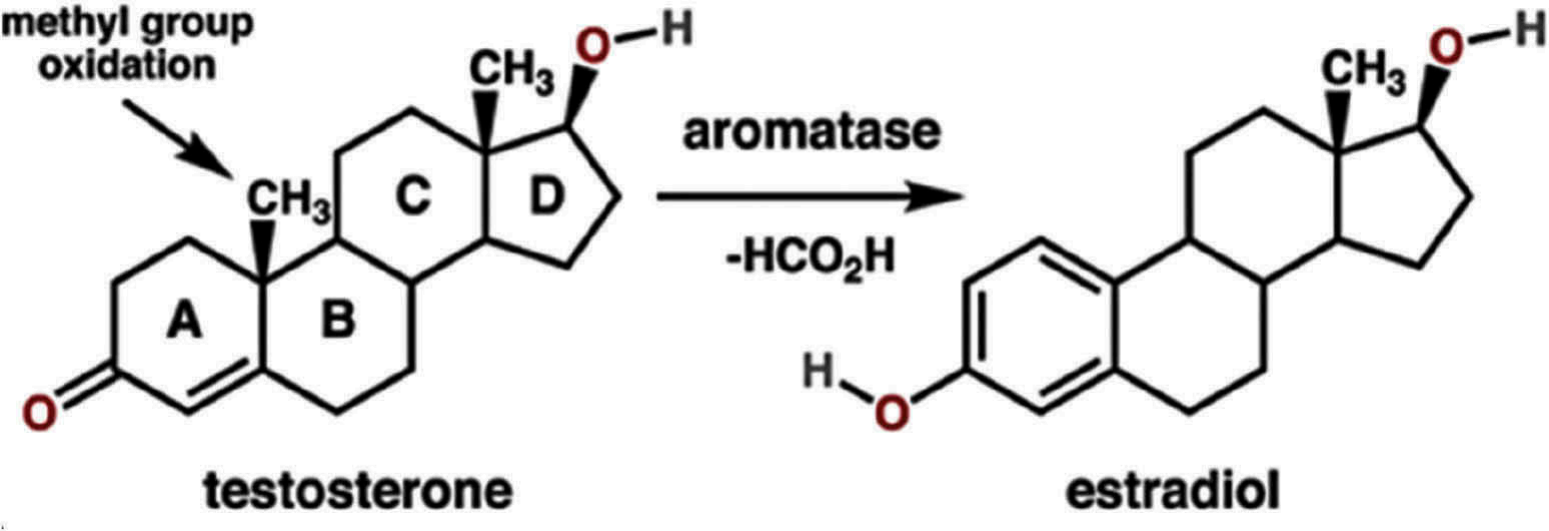
The structure of the main sex-steroids testosterone and estradiol, and the general reaction for the conversion of testosterone to estradiol catalyzed by aromatase. Steroids are composed of four fused rings (labeled A-D). Aromatase converts the ring labeled “A” into an aromatic state. From Wikipedia: aromatase. [19] Author Boghog 2 (own work). Public domain, with thanks.

*Testosterone* is the primary male sex hormone, and an anabolic steroid (Figure 3). In most vertebrates, humans inclusive, testosterone is secreted primarily but not exclusively by the Leydig cells in the testes, and, to a lesser extent, in the ovaries of females. In human males, it plays a key role in the development of the testes and prostate. It promotes the appearance of secondary sexual characteristics such as, compared to females, an increased muscle and bone mass, as well as the growth of body hair. Males being more muscular than females is advantageous in situations in which fighting enemies, and hunting increases the protection and fitness of the family and social group to which they belong. It is a major trigger in generating male-specific behavior. It also plays, among still other functions, a role in preventing osteoporosis, indicating that it exerts some of its effects through the Ca^2+^-homeostasis system. Some derivatives of testosterone also have androgenic activity, e.g. dihydrotestosterone is even more potent than testosterone itself in causing similar effects.

The steroid *Estradiol* (E2), also spelled oestradiol, is the major female sex hormone. Estradiol is produced especially within the follicles of the ovaries, but also in other tissues including the testicles, the adrenal glands, fat, liver, the breasts, and the brain. It is responsible for the development and maintenance of female reproductive tissues such as the uterus, mammary glands, and vagina during puberty, adulthood, and pregnancy. It is not as potent as androgens as an anabolic steroid; hence, females are often less muscular than males. In contrast, in the perspective that females have to be prepared for periods of food scarcity in particular for raising their young/children, the role of estrogens in promoting adipose tissue development is beneficial. It increases not only their own fitness, but also the survival of their young, e.g. through lipid secretion along with milk, a process in which the estradiol precursor progesterone, as well as prolactin, play an important role.

Estrogens are causally related to the appearance of female secondary sexual characteristics such as the breasts, widening of the hips, and a feminine pattern of fat distribution. It is also involved in the regulation of the estrous- and menstrual female reproductive cycles.

### A misconception: the sex-specificity of sex-steroids is not clear-cut qualitative, but only quantitative. Aromatase activity

Both androgens and estrogens are produced in the body of both males and females starting from cholesterol through a series of reactions and intermediates of which the details will not be dealt with here. Thus, their sex-specificity is not a matter of molecular structure, but only a matter of differences in their concentrations in the blood. In adult males, titers of testosterone are about 7 to 8 times higher than in adult females. In females, the opposite situation prevails, but the testosterone titers, although lower than in males are still relatively high in a woman and non-human females[20].. The difference in sex-steroids titers is mainly due to a sex-specific difference in aromatase activity (Figure 3). This enzyme that resides in the membranes of the smooth endoplasmic reticulum (SER), like some other enzymes involved in the biosynthetic pathway of steroids, converts testosterone into estradiol, and androstenedione to estrone. Various factors influence the activity of this enzyme. For example, the anti-Müllerian hormone inhibits its activity.

Aromatase activity is higher in females than in males. One could say that the males get somewhat more exposed (“poisoned”) by testosterone than females, because males have a lower capacity to convert testosterone into estradiol. The opposite holds true for estradiol/estrogens. Although the molecular structures of testosterone and estradiol look alike at first glance, their differential effects on the morphology and physiology are drastic. This follows from their mode of action at the subcellular level.

### Mode of action of sex-steroids: nuclear and membrane receptors

How differential sex-steroid hormonal balances affect every cell of a multicellular organism is often not well understood. One reason is that in contemporary endocrinology the major focus is on control of the expression of steroid hormone-sensitive genes. Such interaction is mediated by nuclear receptors. However, there is a second major target. Through their interaction with membrane receptors sex-steroids also bring about non-genomic effects, e.g. a sex-specific difference in Ca^2+^ homeostasis in all somatic cells of the body. Such effects are often less studied and reported in the endocrine literature. Interaction with membrane receptors yields fast effects (often in seconds), while interaction with nuclear receptors is much slower because it involves protein synthesis.

It is important to keep in mind that both androgens and estrogens are barely soluble in water. The values in males, e.g., are for testosterone 23.4 mg/L at 25°C (PubChem) and for estradiol 3.90 mg/L at 27°C (PubChem). As a consequence, in order to be transported through the bloodstream from their respective sites of synthesis (gonads, adrenal glands, etc.) these hydrophobic steroids need a lipoprotein carrier in the blood that delivers them at the plasma membrane of all cells of the body. Through hydrophobic interactions, the steroid hormones will move from the blood-borne lipoprotein carrier into the lipid bilayer of the plasma membrane. Because all cellular membranes are lipid-rich and fluid, the steroids will start diffusing freely through all connected membrane systems of the cell, and end up in all their membranes: the Rough endoplasmic reticulum (RER), the Smooth endoplasmic reticulum (SER), Golgi, nuclear envelope, mitochondria, etc.. There they may influence many enzymes and signaling pathways, directly or indirectly. The hydrophobic nature of sex-steroids stops them from freely diffusing through the hydrophilic cytoplasm, e.g. to the nucleus, unless they are picked up and transported by a carrier protein with a hydrophobic moiety.

## Physiological effects of sex-steroids

### Gross effects

Even without knowing the details of the interaction of sex-steroids with their receptors, some of the major effects are visible without doing biochemical analyses. As explained above, in both sexes all tissues of the body respond to the sex-specific steroid hormone conditions. In humans, body length, muscle strength, skin properties, distribution and volume of fat/adipose tissue, protein secretion (e.g. through milk production), robustness in time of the skeleton, the types of gametes that are produced, and many more features differ. All these features necessarily depend upon differential protein synthesis (= differential gene activation), and are thus genetically determined. This means that they are governed by the genetic memory system and the central dogma (DNA→ RNA→ Proteins: = Nature), and not by the cognitive memory system (=part of Nurture).

### Effects on Ca^2+^ homeostasis. The Calcigender paradigm

**Figure 4.**
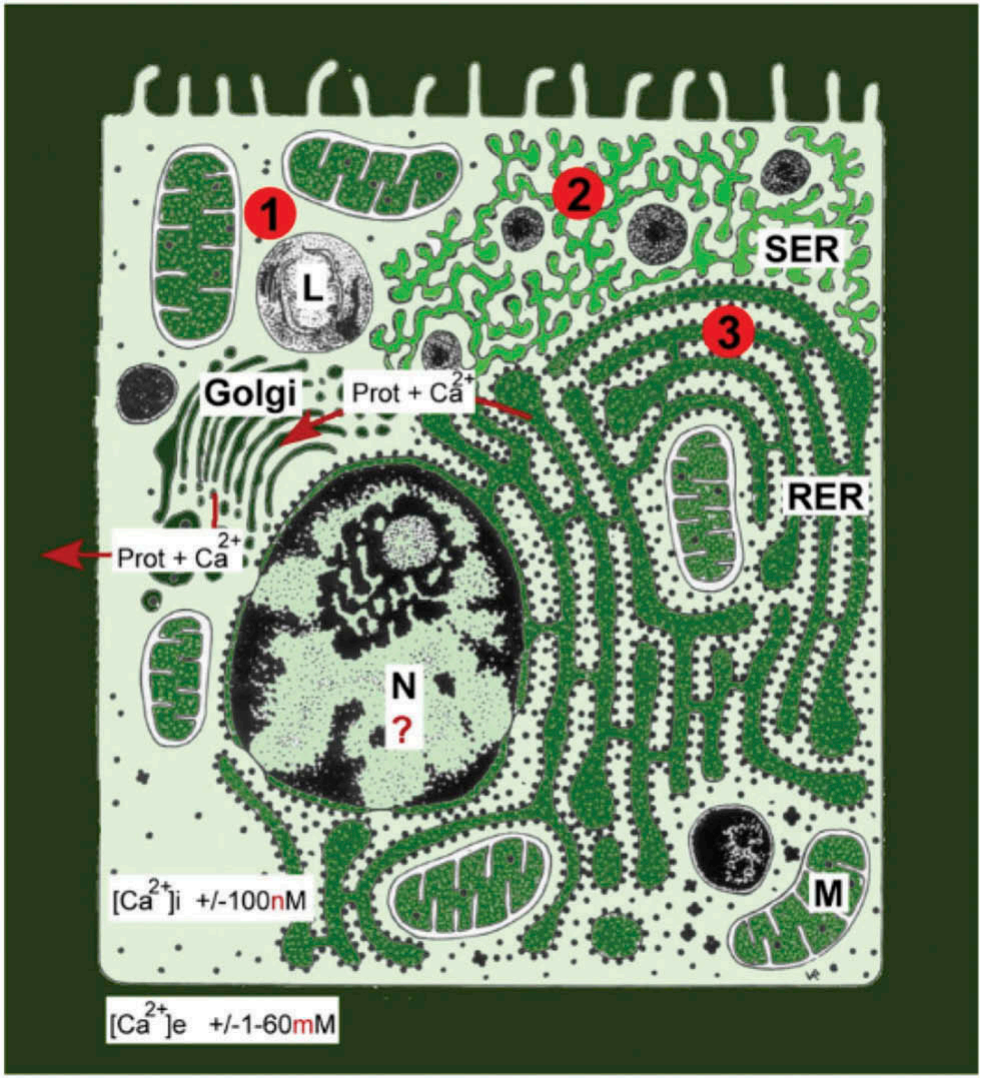
Schematic representation of the main Ca^2+^ gradients in animal cells. From De Loof [7].This figure illustrates that the huge gradients require incessant “efforts” to keep the Ca^2+^ concentration in the cytoplasm at or around a very low concentration of 100 nM. For a more detailed physiological explanation, in particular with respect to the mechanisms indicated by the numbers 1, 2 and 3 see the original Open Access paper[7].

Ca^2+^ is a very potent and ubiquitous ion in all cells, and its concentration is precisely regulated (Figure 4). Behavioral effects are usually fast. If hormonal effects are involved, they are mediated through the interaction of hormones with their plasma membrane receptors, followed by modulation of intracellular pathways. Although the final outcome is that the intracellular Ca^2+^ concentration must be kept very low, particularly in the resting condition of cells, such an outcome can be reached in many different combinations of causal agents. [21] This variability not only yields the outcome that all cells of the body differ in their Ca^2+^ homeostasis system, but that such difference also holds at the organismal level. This is the essence of the Calcigender concept as first formulated by De Loof [22]: males and females, and by extrapolation all gender forms differ in their Ca^2+^-homeostasis which means that there probably are as many gender forms with their specific behavior and physiology as there are sexually reproducing individuals in a species[1].

Out of the extensive literature on this topic, only a few examples will be mentioned. If under the influence of steroid hormones the intracellular [Ca^2+^] rises, cytoskeletal proteins undergo conformational changes. This is well documented in muscle cells, but it also applies to other cell types, in particular, excitable ones. Zylinska et al. [23] investigated the efficiency of selected neurosteroids, and reported that the hormones affect Ca^2+^ transport activity, and that this effect depends on the isoform composition of Plasma Membrane Ca^2+^ ATPases (PMCAs) as well as on the steroid’s structure. PMCAs of which four isoforms occur, keep the free Ca^2+^ concentration in the nanomolar range. Zylinska et al. [24]. also found that in excitable membranes (rat cortical synaptosomes) with a full set of PMCAs, estradiol, pregnenolone, dehydroepiandrosterone apparently increased Ca^2+^ uptake. Calmodulin strongly increased the potency for Ca^2+^ extrusion in (erythrocyte membranes) incubated with 17 beta-estradiol or with pregnenolone. The results indicated that steroid hormones may sufficiently control the cytoplasmic Ca^2+^ concentration within the physiological range.

Calcium ions are essential for proper neurotransmission. Impairment in cytosolic Ca^2+^ concentration and Ca^2+^ signaling disturbs neuronal activity, leading to pathological consequences. [25–27]

### Androgens as anabolic steroids

The higher anabolic effects of androgens over estrogens substantially contributes to sex-specific differences in muscle development and strength. One of the possible definitions of behavior says that “Behaviour is the total sum of all movements an organism makes”. This explains in part the behavioral effects of steroids.

#### Physical training and anabolic steroids mimic each other’s effects: an explanation based upon the principles of Ca^2+^-homeostasis

Human males are in general more muscular, they are stronger, have a bigger heart, more voluminous lungs, more red blood cells and a more robust, and a more stress-resistant skeleton than females. No wonder that some of their activities, and to some extent part of their behavior as well, rely on these effects. In males the average testosterone values fluctuate between 100 and 1000 nanogram/decilitre while in women the normal values are between 10 and 70 ng/dl. This situation applies to many species of placental mammals. It should not be extrapolated to all animal species. In many invertebrates, the opposite situation prevails: in many insect species, females are stronger than males. Here, the sex-hormones are not of the testosterone-estradiol type steroids, but of the ecdysteroid-type, of which the titer is higher in reproducing females than in males. [28]

Skeletal muscle development requires repeated contraction activity. When one breaks an arm that is next immobilized for several weeks in a plaster cast, the arm muscles start atrophying due to the forced inactivity. After the plaster is removed, it takes at least several weeks of training to make the muscles “get stronger” again. How does muscle/body training make the muscle mass increase thereby mimicking the effect of anabolic steroids, and vice versa? Which is the common denominator of physical training and of (administration of) anabolic steroids? At each contraction of a muscle, Ca^2+^ is released from the lumina of the SER of the muscle cells. Reuptake of Ca^2+^ restores equilibrium. Some enzymes needed for steroid biosynthesis reside in the membranes of the SER, others in the mitochondria. At rest, the lumen of the SER is loaded with Ca^2+^ using it as a temporary storage site. High concentrations of intraluminal Ca^2+^ probably inhibit some of the enzymes involved in lipid- or/and steroid biosynthesis. However, in case of muscle contraction during training the Ca^2+^ gradient decreases. Perhaps the short-lived decrease in intraluminal Ca^2+^ is sufficient for lifting the inhibition by the high Ca^2+^ concentration in the lumen of the SER, allowing the synthesis of a small amount of steroids. These steroids then activate the synthesis of muscle proteins (actin, myosin, etc.) stimulating muscular growth. The effectiveness of the intake (oral, injection) of anabolic androgenic sex-steroids competes with that of physical training that causes a moderate local increase (in the muscle cells themselves) of androgenic steroids

#### Progesterone and estrogens are “lipogenic” hormones, in particular in the context of pregnancy

Steroid hormone concentrations in blood (titers) can substantially fluctuate in particular in woman during their menstrual cycle. In many animal species in which reproduction is seasonal, steroid hormone concentrations are linked to particular environmental conditions, e.g. the length of the photoperiod. In general egg, formation involves deposition and accumulation of substantial amounts of yolk material which is rich in proteins, lipids, and glycogen. In Placental mammals with their yolkless eggs, this is not the case anymore. However, the production of milk to nourish the newborn young also requires the mobilization of nutrients, either directly from the ingested food, or from the mobilization of nutrients, in particular lipids, that were stored during pregnancy in adipose tissue. Women normally gain 10–16 kg in weight during pregnancy, a successful strategy in times when food was or is scarcer than it is today in many countries. Such an effect has little to do with Nurture or cognition.

## Steroids, Ca^2+^ homeostasis, Calcigender, cognition, and behavior

### Causal effects between sex-steroids and cognition?

Nurture is intimately linked to learning (imitation, self-learning, teaching, …). Learning implies the presence of a “cognitive memory system”. All cells in both prokaryotes and eukaryotes must have such a system, otherwise they cannot engage in solving problems. [29,30] The cognitive memory system with the self-generated electrical activity of cells as a major foundation, is different from a genetic memory system (DNA → RNA → Proteins). [2,31]

During the pioneering days of experimental endocrine research on the possible influence of sex-steroids and cognition, Christiansen and Knussmann [32] investigated in a group of 11 healthy young men whether a correlation exists between certain cognition activities and titers of testosterone and 5 alpha-dihydrotestosterone in serum and saliva. Several spatial and verbal tests were used. Within the normal physiological range of androgen levels, a positive correlation with spatial ability and field-dependence-independence, and a negative correlation with verbal ability were found. One should keep in mind that a correlation is no proof for a causal relation.

Ulubaev et al. [33]. rightly stated that a distinction should be made between long-term effects of sex-steroids acting through developmental processes and short-term effects acting through learning through the cognitive memory system. The authors reported that although there is convincing evidence that sex-steroid hormones play an organizational role in brain development in men, the evidence for positive effects of sex hormones affecting cognition in healthy men throughout adult life remains inconsistent. To address this issue, they proposed a new multifactorial approach which takes into account the status of other elements of the sex hormones axis including receptors, enzymes, and other hormones. Humans are not an acceptable model for studying the effects of sex-steroids on cognitions because administration of sufficiently high doses of sex-steroids is likely to yield unwanted side effects. Hence, studies were done in humans in which changes in the natural cycles occur, e.g. in menopause, or which were surgically treated. Others were carried out in model placental mammals such as nonhuman primates [34]; or in e.g. rats. [20,35–37] Information was also obtained from studying neurodegenerative diseases, e.g. Alzheimer. [26,27,34]

The question that was asked was whether differences in Ca^2+^ homeostasis can be altered by teaching-learning-imitation? The answer is: no. Perhaps, imposed drastic changes in feeding regime may have some influence on behavior, but this could also cause illness. However, changes in Ca^2+^-homeostasis affecting some aspects of behavior occur during female reproductive cycles (menstrual cycle, pregnancy, breastfeeding, and menopause).

### The sex versus gender issue

**Figure 5.**
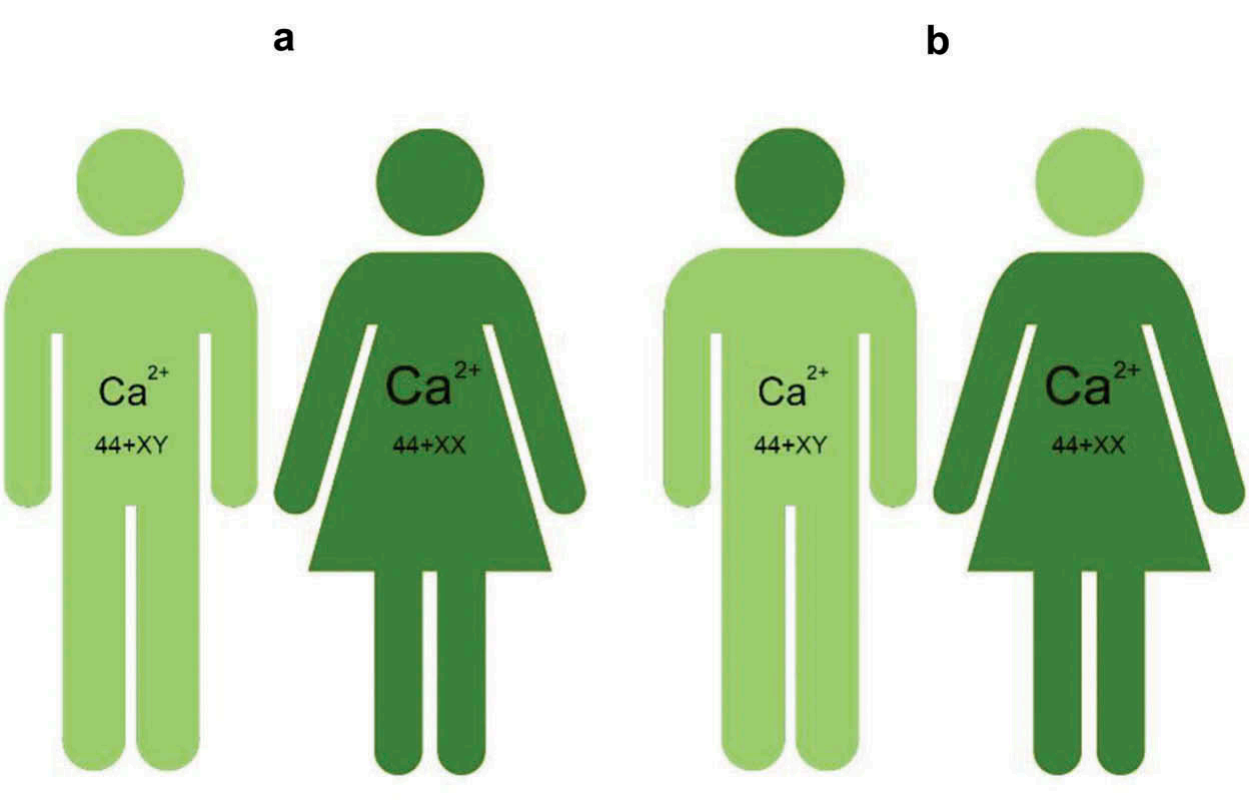
Cartoon illustrating the idea that the main difference between the various gender forms resides in the Ca^2+^-homeostasis system, in particular in some brain areas. Given that the human brain contains about 100 billion nerve cells, it is *de facto* impossible that two individuals have exactly the same Ca^2+^-homeostasis system in the totality of their brain, even if these two individuals are identical twins. This figure illustrates the commonly observed situation that the sexual thinking and behavior of transgenders reflects more the situation of the other heterosexual somatic sex than their own somatic genetic sex. Between these two depicted extremes, numerous intermediate forms are theoretically possible. Indeed, it is more likely that not the whole brain but specific brain regions can display (subtle) changes in Ca^2+^ homeostasis with effects on behavior as a result. Copied from De Loof [1] (own work), no copyright permission required.

This topic, with emphasis on the problem that there are only two sex forms (male and female), but that there are multiple gender forms (Figure 5) has been covered in depth by De Loof [1] and will not be repeated here.

### Sex-gender roles

#### Classical biological roles

In the perspective of the continuation of any heterosexual population over time, the key activity of males and females is the production of gametes. This activity develops very early in embryonic development. It is genetically determined, and it does not require the presence of a partner of the opposite sex. However, in addition to the production of gametes itself, a second indispensable activity is required. Indeed, because gametes are only surrounded by a very thin plasma membrane, they are very vulnerable to harsh environmental conditions, e.g. desiccation. The risks are minimized by a variety of strategies aiming at bringing heterogametes into close proximity as efficiently as possible. In placental mammals and other terrestrial animals, this requires sex-specific types of pre-mating and mating behavior that have to mutually match[1].

#### Social gender roles

As formulated in Wikipedia: Gender role [38], “A gender role, also known as a sex role, is a social role encompassing a range of behaviors and attitudes that are generally considered acceptable, appropriate, or desirable for people based on their actual or perceived sex. Gender roles are usually centered on conceptions of femininity and masculinity, although there are exceptions and variations. The specifics regarding these gendered expectations may vary substantially among cultures, while other characteristics may be common throughout a range of cultures. There is ongoing debate as to what extent gender roles and their variations are biologically determined, and to what extent they are socially constructed.”

A recent example of the belief that gender-neutrality is desirable is the video entitled “Boys don’t cry” published in 2016 by The American Psychological association. The commentary says that ”Growing up, boys often hear “Boys Don’t Cry” as a stereotypical test of manhood. However, this stigmatizes normal human emotions, negatively affecting boys and men. In this short film, we want to change the landscape of emotions for boys and change how masculinity is interpreted – we want to let boys know that it is okay to show emotions”. The ancient Greek difference in the education of boys between Sparta and Athens illustrates that different opinions on such topics already date from at least a few millennia ago.

### Is (more) gender neutrality desirable?

In recent decades the number of efforts to change aspects of prevailing gender roles, which by some groups but in particular by the feminist movement, are believed to be oppressive or inaccurate keeps increasing. [38] A counter-reaction originating from a masculinist movement seems to gain ground. For constructing the respective claims, arguments based on physiological and evolutionary insights are seldom used, a missed opportunity, which is partially corrected in this paper.

From an evolutionary point of view, biologists can only observe that “gender identity” and even more “gender neutrality” [39] is only an issue to a (small) percentage of the individuals of one species, namely the species to which we belong ourselves, the placental mammal *Homo sapiens*. Unanimity about its desirability or as the means to realize gender neutrality is inexistent, to the contrary. To our knowledge, no other species of placental mammals stimulates its young to develop into the direction of gender-neutrality. The reason is simple: in their respective environments, gender-neutrality would very likely result in decreased reproductive fitness. Yet, gender neutrality in its purest form does exist in the animal kingdom, but not in mammals. In some molluscs, namely in some snails, in earthworms, and tunicates hermaphroditism is very common. It is also found in some fish species, less in other vertebrates. In some hermaphroditic snails, a reproducing adult functions as a male for one day, thus transfers sperm to another individual that that day behaves as a female. Another day it turns into a female that can be fertilized by an another individual that behaves that day as a male. This is a consequence of the fact that such animals are hermaphrodites (thus that can produce both eggs and sperm cells, not necessarily at the same moment), but that do not self-fertilize themselves. Self-fertilization decreases genetic variability, hence it should be avoided. Hermaphroditism, although attractive at first sight, is not a major reproduction strategy in the animal kingdom, but in plants it is.

## Discussion

The days that worldwide many people thought, not to say were convinced, that on the average, townsman- and woman were more intelligent that rural ones, that there were racial differences in intelligence, that the classical labor division between man and woman was the normal consequence of only their genetic differences, and most of all, that there are also inborn sex-differences in intelligence between males and females, are largely (not completely) laying behind us. Current school performance of many millions of pupils worldwide proves that the cited “intuitive assumptions” about gradations in “gender performances” (to use a neutral term) were largely Nature-based, thus inborn, need adjustments. The major cause of the change in thinking is the enormous increase in knowledge, both in the humanities and in the exact- and biomedical sciences, as well as the introduction of the term “gender”.

Nowadays “Gender” [40] and “Gender role” [38] are at the very heart of many discussions. This term is very commonly used in the humanities, but much less in classical biology as a discipline of the exact sciences. Fundamental questions asked by biologists are: Why are there only two sexes, one that can produce sperm cells and the other that produces the bigger egg cells, while there are multiple gender forms? ? How does sexual dimorphism come into existence during early development? [41] How was the counterintuitive concept reached that there are as many gender forms as there are gamete producers, and that none is superior over the others? [1,42] Another intriguing question is: How did the heterosexual system of reproduction come into existence in the course of evolution? [3,22] Why did it gradually overrule asexual reproduction, thus reproduction without gametes but that is based upon the principles of regeneration? In contrast, the approach of the humanities focuses much more on the behavioral aspects in gender, in particular on the various aspects of interactions with other individuals with respect to reproduction.

The different “retrograde time perspectives” also matter in the binary Nature versus Nurture debate. In the humanities, specifically in sociology, psychology and education, the focus is on the present day situation. For example, in some countries, the “political scene” gets more and more confronted with issues related to gender, such as equal rights for non-dominant gender forms as well as gender-neutrality in various social environments and the civil status of transgenders. The methods used in the humanities for analyzing a problem barely rely on good knowledge of genetics and of animal physiology. The opposite situation prevails in the biomedical sciences. Here, the contemporary situation in the species *Homo sapiens* is framed in the genetic-, biochemical-physiological, endocrine and evolutionary perspective of the whole animal Kingdom. Thus, a major cause for the lack in unanimity in the Nature versus Nurture debate stems from speaking quite different scientific languages, and using specialized technical vocabularies.

Instead of emphasizing the advantages of division of labor/tasks on the basis of pre-existing genetic differences as was common practice in the past, a recent tendency is towards pushing human society towards gender-neutrality. Gender-neutrality [39] (adjective form: gender-neutral), also known as gender-neutralism or the gender neutrality movement, describes the idea that policies, language, and other social institutions should avoid distinguishing roles according to people’s sex or gender, in order to avoid discrimination arising from the impression that there are social roles for which one gender is more suited than another”. [39] Some positions propagated by some feminist and masculinist groups are controversial.

It is essential that in the Nature versus Nurture discussion one should not only focus on the conscious or unconscious discrimination of a particular gender form for some types of jobs, one should also take into account the reasons why some jobs are more attractive to men, others to women. The factor “I like such job”, is at least as important as “I could do it, if I have no other choice”. A man can become a midwife, but few will spontaneously opt for such job. And young mothers may have a preference for a female midwife. Few woman opt for a job in e.g. road construction, for other reasons than because such a job requires too much muscular labor. Muscular labor is less and less an issue because in complex technology-minded societies machines drastically replaced heavy muscular labor by mainly males. Concurrently intellectual work and activities (the services society) have become more and more important. It gradually results in gender-neutrality with respect to “Who has the capacity can do the job irrespective of the gender issue”. Selective competition with its inherent conflicts may result.

Can Nurture, through all its means instrumental to cultural evolution [31] (many years of education, teaching-learning, imitation, imprinting, forced political or/and social pressure etc.) cause any changes in the genetics, endocrinology, or Ca^2+^-homeostasis of individuals? Unless some body-foreign substances would be administered or without artificial genetic manipulation, the answer is clearly: no. Indeed, the link between sex-specific steroid hormone titers and differential Ca^2+^-homeostasis is so comprehensive that it is biochemically impossible that the cognitive memory system could substantially redirect inborn sex-linked behavior into behavior typical for another gender form. We have to accept that as long as our species will exist, it will continue to carry its physiological, social and psychological evolutionary history. The classical binary approach in which only Nature (genes) and Nurture (education) are more or less equally important in bringing about gender and its behavioral consequences, but in which the Calcigender input is fully neglected, is no longer tenable. The importance of Nature which, among other activities also encompasses the physiology of Ca^2+^-homeostasis and signaling, largely outweighs that of Nurture. It also means that Man is not makeable. He/she is only dirigible in his/her behavior in a limited way. That does not mean that mitigating some “inborn” behavioral traits would a priori be impossible, to the contrary. More gender-neutrality can be advantageous and desirable in some circumstances, but it should not be imposed with approaches that violate the principles of genetics, endocrinology and animal physiology. It can be hoped for that the contribution of biology to the gender-, and Nature-Nurture debates may yield better insights and more tolerance.
